# Why some mothers overestimate birth size and length of pregnancy in rural Nepal

**DOI:** 10.7189/jogh.08.020801

**Published:** 2018-12

**Authors:** Karen T Chang, Luke C Mullany, Subarna K Khatry, Steven C LeClerq, Melinda K Munos, Joanne Katz

**Affiliations:** 1 Johns Hopkins University Bloomberg School of Public Health, Department of International Health, Baltimore, Maryland, USA; 2 The Nepal Nutrition Intervention Project-Sarlahi, Lalitpur, Nepal

## Abstract

**Background:**

Quantitative validation studies alone may not be able to distinguish between instances when participants did not accurately report an event vs when participants did not understand a question. We used an explanatory qualitative study design to acquire an in-depth understanding of why some mothers in rural Nepal overestimate birth size of their newborn and their length of pregnancy.

**Methods:**

We conducted two focus group discussions (FGDs) with study staff who administered a quantitative questionnaire and 12 in-depth interviews (IDIs) with mothers who had participated in a quantitative validation study. Transcripts were coded and analyzed for themes in patterns of meaning within and across FGDs and IDIs. Using this thematic map, we synthesized our data into common and divergent responses from participants to facilitate our interpretation of the quantitative findings.

**Results:**

We identified five themes specific to this analysis: difficulties with the length of pregnancy question, challenges in administering the birth size question, the perceived effect of time since birth on mothers’ ability to remember information, the language and style differences specific to this setting, and the study context shaping the relationship between study staff and mothers who participated and how this may have influenced mothers’ responses. Visual aids may help to scale the question about birth size within a cultural frame of reference for maternal reports to be more interpretable. Among both study staff and mothers, a longer period of time since the birth of a child was thought to be associated with diminished accuracy of maternal reports, a perception not supported by our previously published quantitative findings.

**Conclusions:**

Poor validity of low birth weight (LBW) and preterm birth indicators based on maternal reports may be partly attributed to challenges in maternal understanding of questions assessing birth size and length of pregnancy. Additional research is needed to confirm these findings regarding maternal comprehension and to further evaluate the utility of visual aids developed for this study.

Low birthweight (LBW, <2500g) and preterm birth (<37 weeks) are associated with increased risk of child mortality, severe disability, cognitive impairment, and other long-term health problems [1-3]. Worldwide, about 20 million LBW infants are born annually, and in South Asia, a quarter of all live births are LBW [2,4]. Each year, approximately 15 million preterm newborns are born globally [1,3,5,6]. Preterm birth, disproportionately burdening South Asian and African countries, is the leading cause of neonatal deaths and under-five mortality [5-7]. Some newborns may be both LBW and born preterm, but the conditions are not synonymous as some LBW newborns are small-for-gestational age but term. As part of the Sustainable Development Goals, a target of reducing neonatal and child mortality to 12 and 25 deaths per 1000 live births, respectively, was set for all countries by 2030 [8]. Monitoring LBW and preterm birth indicators over time informs global progress towards achieving these newborn and child health targets [8].

Maternally-reported information collected as part of national household surveys, like the Demographic and Health Surveys (DHS) and the Multiple Indicator Cluster Surveys (MICS), are often the only source of population-based data available on birthweight and preterm birth indicators in low-income countries [9]. Under these approaches, mothers are asked to recall events related to their child’s birth that might have taken place up to five years prior to administration of the survey [9]. Given our reliance on data from such surveys, efforts to evaluate the validity of maternal recall of newborn health are necessary. Quantitative validation studies, however, may not be able to distinguish between instances when participants did not accurately report an event vs when participants did not understand a question [10-12]. Feedback from data collectors who conducted quantitative surveys can be useful to identify any additional probes that were provided to participants who had difficulty understanding a question and to gauge the level of consistency in administering questionnaires across data collectors [13,14]. Prior studies have investigated respondents’ comprehension of survey questions that are similar to those used in DHS and MICS [10-12]. Results of these studies may help to identify questions that may be difficult for mothers to understand and methods that could improve the quality of data collected in surveys. As part of the Improving Coverage Measurement Research Theme, we previously reported results from a validation study of maternal reports of birthweight, birth size and length of pregnancy in rural Nepal [15]. In this paper, using a qualitative explanatory approach, we aimed to acquire an in-depth understanding about how mothers perceive the phrasing of questions assessing birth size and length of pregnancy among a subset of women who overestimated the birthweight and gestational age of their newborns in the validation study. We also describe the experience of study staff in administering these questions, the perceived effect of time since birth on mothers’ ability to remember information, the language and style differences specific to this setting, and the study context shaping the relationship between study staff and mothers who participated and how this may have influenced our findings.

## METHODS

### Study setting

We conducted both the validation and qualitative studies in the rural Sarlahi District of Nepal, where only about half of its predominantly Hindu residents are able to read and write [16]. Over a third of residents are younger than 15 years of age, and almost one in five married women were younger than 15 years old at their first marriage [16].

### Parent trial and validation study

**Figure 1:**
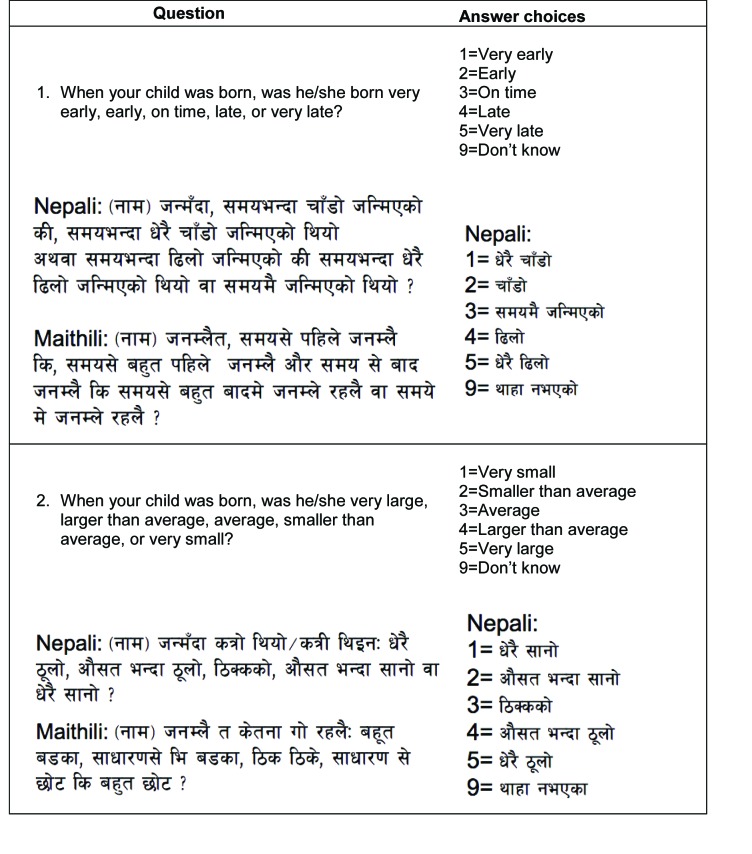
Length of pregnancy and birth size administered in quantitative questionnaire.

The studies were nested within a community-randomized trial that aimed to assess the impact of using sunflower seed oil in full-body newborn massage on neonatal morbidity and mortality (registered at ClinicalTrials.gov (NCT01177111)). The goal of the validation study was to validate postpartum reports of birthweight, birth size and length of pregnancy, by comparing maternal reports directly with data on birthweight and gestational age collected as part of the parent trial. Detailed descriptions of participant selection and analysis for the substudy of the trial can be found in a prior publication [15]. In brief, from April to September 2016, we selected and interviewed 1502 mother/child pairs from the parent trial for one additional follow-up visit to ask mothers to report on circumstances of labor and delivery, immediate newborn care, postnatal care, and neonatal morbidity and care seeking in the first 7 days of life one to 24 months after birth. We compared maternal reports in the validation substudy to prospectively collected data in the parent trial (our “gold standard” estimate) to assess the validity of a) birthweight (<2500 g) and b) birth size (“small” or “very small”) in correctly categorizing newborns as LBW, and c) length of pregnancy (“early” or “very early”) in identifying preterm births. Interviews were conducted in both Nepali and Maithili. In an effort to replicate circumstances of household surveys, we used the Nepali version of the birth size question from the DHS and MICS surveys and translated this question from Nepali to Maithili for use in this study (**Figure 1**). Study staff from the local community were consulted after translation to identify and correct any inaccuracies. This consultation led to a change in the Maithili version of the birth size question used in this study, which employs language more specific to the dialect spoken in Sarlahi District while the DHS Maithili version includes phrases from the dialect in the Janakpur region to the east of Sarlahi. We modeled the length of pregnancy question after the birth size question in English and then translated the question to Nepali and Maithili (**Figure 1**), and the local study team also provided input to refine translations in both Nepali and Maithili. We observed low individual-level accuracy and high population-level bias for all three indicators, motivating this qualitative approach to explore maternal understanding of questions administered.

### Qualitative study

In the validation study, we concluded that maternal reports underestimated LBW and preterm birth, particularly in a setting with relatively high prevalence of both [15]. Our results also showed time since birth did not affect the validity of maternal reports [15]. The objective of this qualitative study was to explain and seek a better understanding of the quantitative results of the validation study, that is why some women may have overestimated the birthweight or gestational age of their newborn. To do so, we identified questions of interest from the follow-up questionnaire based on feedback from study staff during data collection supervision visits and on preliminary analyses of quantitative data, focusing on discordant results between maternal reports and gold standard data on LBW and preterm status.

Our explanatory qualitative approach incorporated perspectives from both study staff who had administered questionnaires and participants in the validation study. In August 2016, during the last month of data collection for the validation study, we conducted focus group discussions (FGD) with the study staff who had administered the quantitative form. To ensure only study staff who wanted to participate were recruited and others did not feel coerced, we limited the number of FGDs to two. The study staff were relatively homogeneous in terms of socioeconomic status, all were local residents, female, and all received standardized training on administration of the quantitative questionnaire. For these reasons, we felt that two FGDs were sufficient to capture the information of interest. A discussion guide was created to cover the following themes: reflection on experiences with administering the quantitative form, identification of questions mothers had difficulty answering, discussion of reasons difficulties were encountered, description of probes used for clarification, and suggestions for how questions could be improved for better understanding. FGDs included a more focused discussion about the birth size and length of pregnancy questions but also allowed for study staff to discuss other questions of the quantitative survey they thought were difficult for mothers to answer. FGDs were conducted by locally-resident, female qualitative interviewers who were fluent in Nepali and Maithili, from the same community as our study staff, and in non-supervisory roles in an attempt to allow study staff to more openly share their experiences working on the study. We conducted the FGDs in a private room at one of the field offices.

Based on information from the FGDs, we identified and developed visual aids for use during in-depth interviews (IDI). Because the explanatory design of this qualitative study aimed to better understand why some mothers may have overestimated the birthweight or gestational age of their newborn, those who provided discordant responses in the quantitative validation study were selected for IDIs. Mothers residing in these areas who responded discordantly to at least three of the survey questions of interest relative to gold standard data from the parent trial were eligible to participate in IDIs. From September to November 2016, qualitatively-trained, local female interviewers (literate with no more than a high school level education), who were different from those who conducted the quantitative questionnaires of the validation study, administered oral consent in Nepali or Maithili and obtained a signature or thumbprint for mothers who agreed to participate. IDIs were conducted one-on-one in a private area in households of participants. An interview guide was created to cover the following topics: willingness to discuss labor and delivery and newborn health, attitudes about newborn health checks, views about whether time since birth affects mothers’ ability to remember what happened, and reflection on questions that generated discordant responses and methods to improve the accuracy of maternal responses.

Discussion and interview guides were created in English and translated into Nepali and Maithili by local staff. Debrief sessions were conducted with qualitative interviewers following FGDs and IDIs to reflect on the quality of the discussion/interview, summarize content, edit questions for understanding, and discuss challenges. FGD and IDIs were audio recorded and transcribed from Maithili to Nepali by the interviewers. Supervisory staff fluent in Maithili and Nepali in Sarlahi reviewed the first transcript completed for each translator for quality control. The Nepali transcripts were then sent to translators in Kathmandu for translation to English. Supervisory staff fluent in Nepali and English in Kathmandu also reviewed the first transcript completed for each translator to check for quality. For any additional clarifications that were needed in the English versions of transcripts, another native Nepali speaker fluent in English reviewed Nepali versions and re-translated sections as needed. Recordings, transcripts, and translations were all de-identified.

### Ethical approval

The parent trial and the substudy, including the qualitative component, both received ethical approval from the Johns Hopkins Bloomberg School of Public Health Institutional Review Board, Baltimore, MD, USA. Local approval was received from the Tribhuvan University Institute of Medicine, Kathmandu, Nepal for the parent trial and from the Nepal Health Research Council, Kathmandu, Nepal for the substudy.

### Data analysis

This analysis focused on questions related to birth size and length of pregnancy and the effect of time since birth on mothers’ memory. We analyzed transcripts using Atlas.ti Scientific Software. We generated hypotheses based on preliminary analyses of quantitative data from the validation study focusing on birth size and length of pregnancy and the interview guides, which deductively informed the development of codes in an initial codebook. In a first round of coding, we both applied initial codes and inductively added new codes based on additional themes that arose. In a second stage of the coding process, all codes were grouped into overarching axes and refined to create a final codebook, which was applied to a second review of transcripts. We used thematic analysis to search for patterns of meaning within and across FGDs and IDIs [17,18]. Using this thematic map, we synthesized our data into common and divergent responses from participants to facilitate our interpretation of the findings [17,18].

## RESULTS

**Figure 2:**
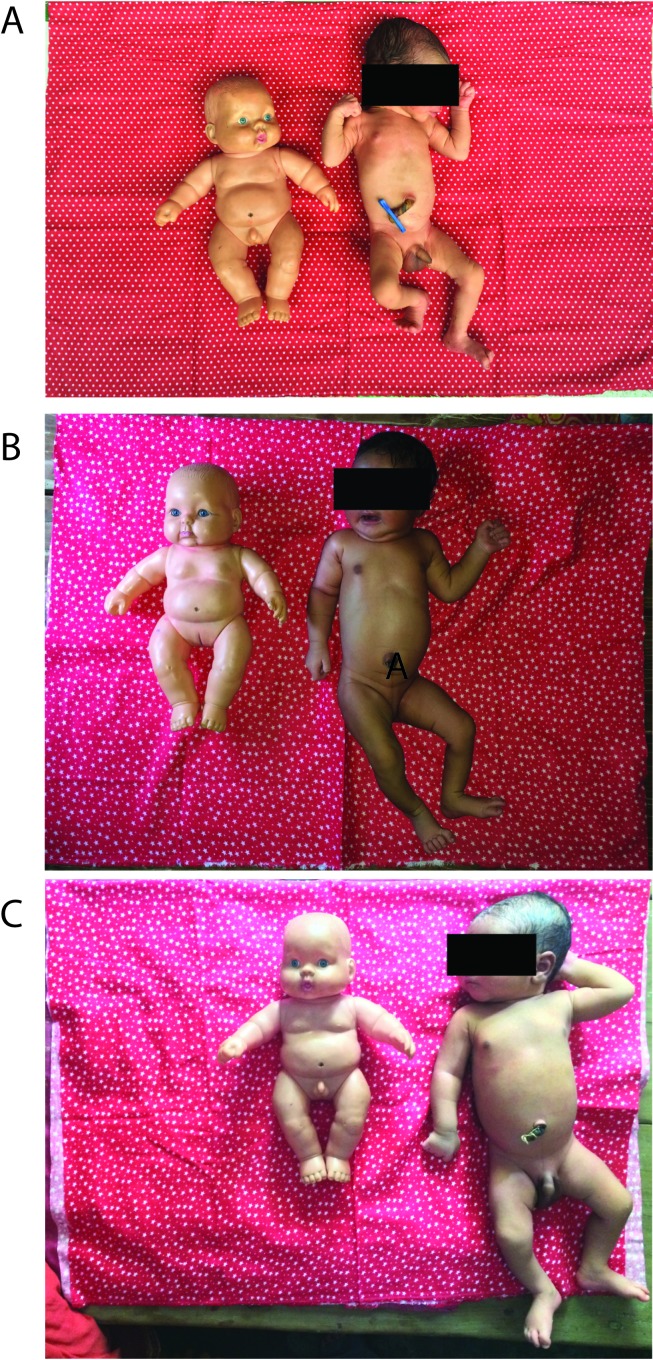
Visual aids for birth size question.

We first conducted two FGDs, each with 6 study staff, who had administered the quantitative form. All study staff were female, had at least a high school diploma and ranged from 20 to 50 years of age. In the first FGD, four of our study staff were of the Madhesi ethnicity and two were Pahadi. In the second FGD, two were Madhesi and four were Pahadi. **Figure 2** shows the dolls and photos of newborns of different sizes (A-2.2kg, B-2.6kg, C-3.1kg) developed based on suggestions from study staff during FGDs and used in administering questions related to birth size in IDIs. The dolls were used to anchor the size of newborns to allow for comparison across photos, and not intended to indicate a Nepali newborn. The dolls, also used during health visits sponsored by government programs in the district, were familiar to participants and also presented to mothers during IDIs for reference. **Table 1** describes the characteristics of the 12 mothers who were selected for IDIs, conducted after completion of FGDs. Five themes emerged in the analysis within and across FGDs and IDIs: difficulties with the length of pregnancy question, difficulties with the birth size question, the effect of time since birth on mothers’ memory, the language and style differences specific to this setting, and the relationship between staff and mothers in the quantitative interviews.

**Table 1:** Characteristics of mothers interviewed in qualitative follow-up

Characteristic	n (%)
**Child’s age at the time of administration of the quantitative form**	
<12 months	5 (42)
≥12 months	7 (58)
**Child sex:**	
Male	7 (58)
Female	5 (42)
**Place of delivery:**	
Home	4 (33)
Facility	8 (67)
**Maternal age:**	
<20 years	4 (33)
≥20 years	8 (67)
**Maternal education:**	
No schooling	4 (33)
Any schooling	8 (67)
**Parity:**	
Primiparous	6 (50)
Multiparous	6 (50)
**Ethnicity:**	
Madhesi	12 (100)

### Difficulties with the length of pregnancy question

Study staff reported encountering difficulties in maternal understanding of the length of pregnancy question during both FGDs. Citing that the question was too long and the phrasing of the question was confusing, staff in an FGD also suggested that mothers might require more context to understand what the word “time” referred to as the word was used repeatedly in Nepali and Maithili translations of the question. From staff experiences, mothers occasionally misunderstood the question as asking about the time of day the child was born or the length of time they were in labor, as described below:

Participant 2: When we ask about the time, they think we mean morning, afternoon, or night.

Participant 3: That’s what happened when we asked this question. (Laughing)

Participant 2: Yeah, some say it happened in the morning, others at night.

Participant 4: That’s what they immediately understand by time.

Participant 2: They specify that evening is a time, too, and that it didn’t happen at night.

Participant 3: Like Participant 1 said (pointing at Participant 1) actually, women remember the bits starting from their labor pain, and their attention is stuck there.

Moderator: Hmm…

Participant 3: And that’s probably the reason why it’s difficult for the mothers to answer the question- (Participant 2 interrupting)

Participant 2: When we went for training, we were instructed what the phrase “before time” means in the question. But usually, the mothers don’t know what the phrase “before time” means. They don’t know what ‘time’ is referring to. They don’t know which “time.” Maybe that’s why they get confused.

When asked about how the question could be rephrased for better comprehension and what additional probes study staff would use to help mothers understand, FGD participants suggested specifying ‘preterm’ or ‘due date’ in the following discussion:

Participant 3: When the child was born, was he born at term or preterm… I don’t think it is right. (Laughing)

Participant 4: But this is how we explained in the field. We ask them if the child was born at term or prematurely and explain to take the difference between months and days.

Participant 6: If the baby was born prematurely- (Participant 1 interrupted)

Participant 1: Was your baby born after the time or before it?

Participant 6: We asked like that.

Participant 4: They don’t understand like that. (Speaking to Participant 1) Was the baby born right at the due date or before it? Or was the baby born way before the due date or past the due date? Or way past the due date or right on time? That’s how we asked and they understand it easily.

Data from IDIs with mothers supported these findings. When asked about the length of pregnancy using the original phrasing of the question, one mother described the time of day her child was born and made reference to the possibility of requiring a caesarean-section had her child not been born within a specified time since labor:

Participant: The doctor said that if [I] deliver the baby at 8 o’clock, then it is okay.... The baby was not born at 8 o’clock; she was delivered at 7 o’clock. (21 year old mother)

When asking this same mother the question specifying ‘preterm’ as an example of ‘early and specifying ‘overdue’ as an example of ‘late,’ the mother responded, her “baby was born at nine months.”

### Difficulties with the birth size question

Study staff were instructed to ask the birth size question exactly as phrased in the Nepali DHS and MICS surveys. Some difficulties encountered in administering this question included unfamiliarity with the Maithili word for “average” (“ausat”) and using the Maithili word for “normal” (“samanya”) size in clarifying probes.

Participant 3: Maybe because they don’t understand the word “ausat” that they don’t get the rest. Instead of the word “ausat,” when we asked them how was the baby, big, small, average, in their way, they understood it.

Participant 2: Yes. When we said smaller or bigger than average, they’d understand immediately. When asked whether [the baby] was bigger or smaller than was supposed to be, they said that it was average [“samanya”- the Nepali word for normal]. The answer comes that way…

Moderator: Yeah… In Participant 2’s (pointing at Participant 2) opinion, they wonder what the word means and how they should respond. Similarly, are there any other- (Participant 4 interrupts)

Participant 4: When we say ‘ausat’ means average, they don’t understand but when we just say “average” [“samanya”- the Nepali word for normal] they understand.

In another FGD, staff explained that the question was sometimes misunderstood as asking about the height or length of the child, rather than the weight.

Participant 6: When we ask how big was the baby then the first thing that comes to their mind is the height of the child and not the weight and therefore it becomes necessary to repeat the question indicating that it is related to weight.

When asked how mothers’ understanding of this question could be improved, study staff suggested using colored photos of newborns of different sizes to show to mothers for a frame of reference.

Participant 6: One picture/photo will not do anything. (P6 and P3 agreeing to the statement). Rather a picture of a fat child and a picture of a thin child might have been helpful. It might be helpful if the pictures were in color.

Moderator: So you think that the photo of a fat or thin child will be helpful in understanding this question?

(Participant 3 and Participant 6 speaking together) The photo of newly born children in color will be better for understanding of the mothers.

Based on this suggestion from the study staff, we created the visual aids in **Figure 2** to use in IDIs with mothers. When our qualitative interviewers asked mothers about their child’s size at birth, a common response was, “My child was neither too big nor too small; he was normal.” As a follow-up question, when asking a mother of a child, who was born at home, and weighed 2.25kg at birth, to then select a photo of a newborn whose size most closely resembled that of her child when he was born, one mother “looked at all the photos and then took one of the photos in her hand that was the smallest in size (Photo A) and said that her child was like that photo but was thinner than the photo.” In further discussion, the mother again described her child as being of average size, “As I told you, my child was neither very big nor very small. My child was somewhere in between.” Although the reported birthweight and birth size were both overestimated, the selected photo provided a closer estimate of the child’s size.

#### Effect of time since birth on mothers’ memory

Some of the study staff thought the length of time since birth did affect the accuracy of mothers’ responses in some cases.

Participant 6: The women who have a five- or six-month old child would remember. But at the beginning of data collection, there were women who had thirteen-month old children, and it was hard for them. They had even forgotten the answers to some of the questions. Others were all right.

Other staff pointed out that maternal accuracy depended more on the individual ability of the mother to remember things.

Participant 3: So some of the women remember things even after two years while some others do not remember the things that happen within a month or so.

Participant 2: Yes, they do not remember.

Participant 6: This is problem of some women. All the women do not have the same memory power.

Participant 5: All people do not have same type of brain.

This question was also posed to mothers during IDIs, and many mothers noted that their memory of events may fade with time. One mother said that day-to-day obligations and worries prevent her from remembering events at birth.

Participant: If the mothers are free from other things and keep on thinking the same thing again and again then they can remember it. Therefore if the mothers have time to think on the time of their delivery and the baby conditions at that time then there is every chance that they can remember for longer time. But they have to engage themselves in so many other domestic chores like how to get baby educated, how to earn both times meal for family, looking after the animals and small children etc. So, most of the time their mind is occupied with these things. Don’t you think that these are more important to spend time on rather than just thinking over and over about their delivery time? (19 year old mother)

### Language and style differences

A frequent theme that emerged from our FGDs with study staff was the distinction between the Nepali and Maithili language and styles. In discussions related to the length of pregnancy question, one staff member referred to using the Maithili language to aid mothers’ understanding, saying “Only some [mothers] won’t [understand]. The mothers will understand if we explain in their language.” Another staff member in a different FGD said, “As long as we explained the question in their style (Maithili), they understood it at once, and we didn’t have to probe a lot.” In discussions related to misunderstanding of the birth size question, one of our staff explained that in “Maithili society,” mothers frequently thought the question was asking about height or length rather than weight.

### Relationship between staff and mothers

In both FGDs, there was a perception that literacy and education levels of mothers were linked to the ability to understand questions. In reference to the birth size question, one of the staff explained, “When we meet literate women, when we say bigger than average and smaller than average, they understand it right away. But when we meet others, they don’t know what it means.” Later in this same discussion, another staff member shared her experience during household visits, “In fact when we go [to their houses], mothers are a bit intimidated and feel shy to talk to us.” While providing suggestions for props and pictures to use as visual aids with mothers during IDIs, study staff made a distinction between mothers who are “smart” vs “silent.”

Moderator: Let’s say (showing a doll) this one here. If you question the mothers showing this doll… If you use this doll to question the mothers, what do you think will happen?

Participant 3: You have to show this to silent mothers and question.

Participant 4: (Using the doll) This is how the navel was examined for any signs of danger in the body of the child- (P2 interrupting)

Participant 2: If that’s the case, they won’t understand our questions at all. And if she’s silent there will be no interview. (Laughing and P4 joins)

Moderator: Yeah…

Participant 2: You have to show it to a mother who is smart rather than a mother who is silent.

The theme of being educated vs uneducated was also reflected in IDIs in responses from mothers related to the effect of time since birth on maternal recall.

Participant: Sometimes the things are remembered.

Interviewer: They are remembered?

Participant: No, it is not all remembered. It is not written down like how the educated people do it. Nobody can remember everything. (21 year old mother)

When asked to explain how one mother understood the question about length of pregnancy, she pointed out the difference in literacy and education between herself and the qualitative interviewer.

Interviewer: How do you understand this question? What do you think this question is trying to ask?

Participant: What you asked me is… See, you are educated and I am illiterate. Despite that I have to use whatever wits I can gather to work. Say, I have to think about what is good and what is bad. I have to find a good path. (24 year old mother)

## DISCUSSION

We previously reported low accuracy in maternal reports used to calculate LBW and preterm birth indicators as compared to birthweight and gestational age data collected as part of a community randomized trial in rural Nepal [15]. In this study, we sought to identify possible reasons for some mothers to overestimate the birthweight or gestational age of their newborns. Based on qualitative results from FGDs with study staff and IDIs with mothers who participated in the quantitative component of this study, low accuracy of maternal reports may be partly attributed to inconsistent understanding of questions related to birth size and length of pregnancy among mothers in rural Nepal. Although we had translated our quantitative forms to both Nepali and Maithili and the local study team reviewed translations, our study identified challenges in the phrases used, and style and the length of questions administered. While we had adopted the Nepali version of the birth size question verbatim from the DHS and MICS surveys, we had created a Maithili version of the question for use in this study. Supplementing the birth size question with visual aids facilitated more accurate assessments of birth size. The length of pregnancy question was developed using a similar sentence structure as the birth size question in English. However, from this analysis, we observed that poor translations in both Nepali and Maithili largely affected mothers’ ability to accurately describe the length of pregnancy. In a study assessing the comprehension of questions in a Tanzania AIDS Indicator Survey, Yoder and Nyblade describe difficulties encountered with translation from English to Kiswahili, including problems with style and structure [12]. The authors encouraged the use of translations that are not literal, but rather, reflect the original intent of the question. Cognitive interviews may be a useful tool to gauge participant comprehension following translation of a survey from English into a local language [19-21]. Other approaches that ask different types of questions and use simplified sentence structures to measure the same construct across different cultures may also be necessary [22]. Creating a template of questions in English and simply translating them into other languages may fall short in guaranteeing equivalence in what is measured across study settings and may not be sufficient in ensuring data quality [23]. This study demonstrates the need to translate surveys into local languages and to ensure questions have equivalent meaning even though these procedures may be resource intensive and may complicate survey implementation.

To aid maternal understanding of the birth size question, we asked mothers during IDIs to refer to photos of newborns of varying weights and identify one that most resembled the size of her child at birth. We observed that while mothers with LBW babies frequently described their child as being of ‘average’ or ‘normal’ size at birth without the visual aid, mothers often selected the photo of the smallest child. Channon describes the influence of various community and regional factors within a societal context on mothers’ perception of birth size that shape a point of reference for how they assess their child’s size [24]. Relative to the global context, newborns in this rural Nepali setting are generally smaller, perhaps influencing mothers to perceive smaller children as being of average size. Visual aids may assist in scaling the question about birth size within a cultural frame of reference for maternal reports to be more interpretable.

Interestingly, both study staff and mothers believed accuracy of maternal report would diminish over time, consistent with our initial hypothesis; however, our quantitative findings do not support this theory in the context of generally poor maternal accuracy, even at one month after birth. Previous research has found greater accuracy and agreement between maternal reports of birthweight and gestational age and medical records associated with shorter periods of recall [25-27] while others have observed no difference over time [28-30]. However, comparability to our study is limited since these studies assessed reports following longer periods of time over years rather than months.

We reflect on the relationships between the study staff of the quantitative validation study and mothers who participated in quantitative interviews. Study staff had been recruited from the same local community in an effort to facilitate more open communication with participants. However, we observed a consistent thread in both our FGDs and IDIs that suggested a power dynamic existed between the study staff and the participant that likely influenced the types of responses collected in our study [31]. Overall, study staff of the validation study were viewed as being educated and literate while mothers who had difficulty answering questions were considered uneducated and illiterate. Qualitative interviewers were also locally-resident workers who were trained to first establish a rapport with participants at the start of interviews, so that women would feel more comfortable before being asked interview questions. However, in a community with low literacy levels, those who are literate and able to read a questionnaire aloud may be perceived as being of higher status. This dynamic may have precluded mothers from being more open in sharing their opinions because they felt intimidated or shy. It is likely that this same dynamic is operating in DHS and MICS in many countries where literacy in rural areas is low.

Finally, there were several limitations in this study. Although we identified repeat concepts and ideas related to difficulties with the length of pregnancy question, difficulties with the birth size question, the effect of time since birth on mothers’ memory, the language and style differences specific to this setting, and the relationship between staff and mothers in the quantitative interviews, logistical circumstances precluded data collection to continue until saturation; therefore, other contributing factors for the discordant maternal responses may exist. However, we believe that the combination of results from the FGDs and the IDIs do contribute to an understanding of further work to explore how some of these DHS questions may be altered in the future for increased fidelity. Transcripts were subjected to several layers of translation. Qualitative interviewers listened to audio recordings of the interviews that were primarily conducted in Maithili and directly translated these into Nepali, which may have resulted in a loss of emic terms. Transcripts were subjected to quality control procedures, where supervisory staff fluent in both Nepali and Maithili checked the first transcript completed for each translator. Although clarifications were sought from a native Nepali speaker during analysis of English transcripts, the author is a non-native Nepali speaker, further limiting our findings from this analysis.

## CONCLUSIONS

Poor validity of LBW and preterm birth indicators based on maternal response may be partly attributed to challenges in maternal understanding of questions assessing birth size and length of pregnancy. Findings from this qualitative study suggest specific terms in Maithili translation and sentence structure affected maternal comprehension. Visual aids, like pictures of newborns of varying sizes, may help to scale maternal perception of birth size in specific settings. In addition, relationships and dynamics between interviewers and participants may affect the nature of responses. More work is required to further explore maternal comprehension of these questions in similar rural and low-income settings as a prelude to improving content and context in DHS/MICS surveys.
